# Rupatadine Protects against Pulmonary Fibrosis by Attenuating PAF-Mediated Senescence in Rodents

**DOI:** 10.1371/journal.pone.0068631

**Published:** 2013-07-15

**Authors:** Xiao-xi Lv, Xiao-xing Wang, Ke Li, Zi-yan Wang, Zhe Li, Qi Lv, Xiao-ming Fu, Zhuo-Wei Hu

**Affiliations:** Molecular Immunology and Pharmacology Group, State Key Laboratory of Bioactive Substance and Function of Natural Medicines, Institute of Materia Medica, Chinese Academy of Medical Sciences & Peking Union Medical College, Beijing, P. R. China; University of Pittsburgh, United States of America

## Abstract

A similar immune response is implicated in the pathogenesis of pulmonary fibrosis and allergic disorders. We investigated the potential therapeutic efficacy and mechanism of rupatadine, a dual antagonist of histamine and platelet-activation factor (PAF), in bleomycin- (BLM-) and silica-induced pulmonary fibrosis. The indicated dosages of rupatadine were administered in rodents with bleomycin or silica-induced pulmonary fibrosis. The tissue injury, fibrosis, inflammatory cells and cytokines, and lung function were examined to evaluate the therapeutic efficacy of rupatadine. The anti-fibrosis effect of rupatadine was compared with an H1 or PAF receptor antagonist, and efforts were made to reveal rupatadine’s anti-fibrotic mechanism. Rupatadine promoted the resolution of pulmonary inflammation and fibrosis in a dose-dependent manner, as indicated by the reductions in inflammation score, collagen deposition and epithelial-mesenchymal transformation, and infiltration or expression of inflammatory cells or cytokines in the fibrotic lung tissue. Thus, rupatadine treatment improved the declined lung function and significantly decreased animal death. Moreover, rupatadine was able not only to attenuate silica-induced silicosis but also to produce a superior therapeutic efficacy compared to pirfenidone, histamine H1 antagonist loratadine, or PAF antagonist CV-3988. The anti-fibrotic action of rupatadine might relate to its attenuation of BLM- or PAF-induced premature senescence because rupatadine treatment protected against the *in vivo* and *in vitro* activation of the p53/p21-dependent senescence pathway. Our studies indicate that rupatadine promotes the resolution of pulmonary inflammation and fibrosis by attenuating the PAF-mediated senescence response. Rupatadine holds promise as a novel drug to treat the devastating disease of pulmonary fibrosis.

## Introduction

Pulmonary fibrosis is a chronic interstitial lung disease, which can be induced by a diversity of insults, including microbial infection, smoke, chemical materials, and environment contamination, and is a major structure basis for many chronic fibroproliferative lung diseases [1,2]. The mechanism responsible for the pathogenesis of pulmonary fibrosis remains unclear. Although immunosuppressive agents have been recommended as a therapeutic regimen, the anti-fibrotic efficacy of this strategy is limited [3]. Thus, there is an urgent need for developing new anti-fibrotic therapeutics for these fibroproliferative lung diseases.

Many studies demonstrate that tissue fibrosis is mainly driven by chronic inflammation and that the type of immune response is a critical factor affecting the pathogenesis of pulmonary fibrosis [4]. For instance, the Th2-type immune response critically contributes to the development of pulmonary fibrosis by suppressing the resolution of inflammation and promoting tissue repair [5], whereas the Th1-type immune response attenuates the development of pulmonary fibrosis by promoting the resolution of chronic inflammation [6]. The Th17-type immune response also participates in the pathogenesis of pulmonary fibrosis by a mechanism that is similar to the Th2-type response [7]. Recently, IL-10, a regulatory T cell (Treg) cytokine, has been found to trigger pulmonary fibrosis [8]. Therefore, the manipulation of immune responses may be a promising therapeutic strategy for the prevention and treatment of pulmonary fibrosis [7].

Interestingly, the Th1/Th2 paradigm not only affects the development of tissue fibrosis but also contributes to the development of allergic diseases [9]. For instance, Th2-type cytokines, such as IL-4, IL-5, and IL-13, are critically involved in all aspects of the development of allergic diseases [10]. Recent studies have also demonstrated that Th17 cells and IL-17 may participate in the pathogenesis of allergic diseases by their regulation of innate immunity [11]. Conversely, increases in the populations of lung-infiltrating allergic cells, including mast cells, eosinophils, basophils, and the release of allergic mediators from these cells, contribute to the development of pulmonary fibrosis [12–15]. These observations suggest that anti-allergic drugs may have therapeutic potential against pulmonary fibrosis.

In this study, we investigate the potential efficacy and mechanism of rupatadine in the prevention and treatment of pulmonary fibrosis. Rupatadine is a dual antagonist of H1 and PAF receptors. Rupatadine has been prescribed for the treatment of allergic rhinitis and chronic urticaria [16]. A number of studies have shown that rupatadine can inhibit the degranulation of allergic cells to block the release of allergic mediators from these cells [17]. We find that rupatadine can attenuate acute and chronic pulmonary fibrosis, improve lung functions, ameliorate inflammatory responses, and decrease fibrotic animal death. Our studies indicate that the therapeutic role of rupatadine against pulmonary fibrosis may be due to its antagonism of PAF-mediated p53/p21-dependent cellular senescence, both *in vivo* and *in vitro*.

## Materials and Methods

### Animals and reagents

Male C57BL/6J mice (18 g, 6–8 wk) and Sprague-Dawley rats (180-200 g) were obtained from Vital River Laboratory Animal Technology (Beijing, China). All animal protocols conformed to the Guidelines for the Care and Use of Laboratory Animals approved by the Animal Care and Use Committee of Chinese Academy of Medical Sciences and Peking Union Medical College. Bleomycin (BLM) was purchased from Nippon Kayaku (Tokyo, Japan). Silica, Histamine and C-PAF (Carbamyl-PAF, a non-hydrolyzable bioactive analog of PAF) were purchased from Sigma-Aldrich, Inc. (St. Louis, MO). Rupatadine was a generous gift from Zhejiang CiFu Pharmaceutics Inc. (Zhejiang, China) and was dissolved in distilled water and administered by oral gavage. Pirfenidone was purchased from Shanghai Richem International Co (Shanghai, China). Loratadine was purchased from National institutes for Food and Drug Control (Beijing, China). CV-3988 was purchased from ENZO life sciences (NY, USA). Histamine and PAF ELISA kits were obtained from Cusabio Biotech Co., Ltd (Wuhan, China), while the other ELISA kits were purchased from eBioscience (San Diego, CA). Alexa Fluor 488, 549 and 633 were obtained from Invitrogen (San Diego, CA). Anti-mouse **α**-SMA, E-cadherin, vimentin, p53, pp53, pRb, ppRb, LC3, beclin-1, lysosome-associated membrane protein 1 (LAMP-1), p62, mTOR, pmTOR, NF-κB, pNF-κB, STAT3, pSTAT3, and GAPDH Abs were obtained from Cell Signaling Technology (Danvers, MA). Anti-mouse collagen I, IL-1α, IL-1β, IL-6, IL-8, IL-10, IL-17, TNF-α, TGF-β, γ-H2A.X T-bet, and GATA3 were purchased from Abcam Inc. (Cambridge, UK). Anti-mouse C/EBP-β, MCP7, iNOS, Arg-1, MAC3 were purchased from Santa Cruze Biotechnology, Inc. (Santa Cruz, CA).

### Generation of pulmonary fibrosis and therapeutic protocol

Pulmonary fibrosis was generated as previously described [7]. In brief, the mice were anesthetized with 50 mg/kg pentobarbital i.p. (Merck). Using an insulin syringe, 50 μl of LPS-free saline clinical grade BLM (5.0 U/kg, and this dose of BLM could maximize detection of survival differences) was injected directly into the trachea. The mice were treated with rupatadine (1.5, 3, 6 mg/kg once a day, equivalent to 8, 16, and 32 mg of rupatadine fumarate /day for human dosages which were calculated by body weight and body surface area) by oral gavage from day 10 to 28 after BLM administration. Mice in sham and model groups received an equivalent volume of water. A pirfenidone dose of 400 mg/kg was given once a day by oral gavage from day 10 to 28 after BLM administration. To determine the role of H1 and PAF receptor in pulmonary fibrosis, the mice were treated with loratadine 3 mg/kg per day (o.g.), and/or CV-3988 3 mg/kg per day (i.p.) from day 10 after BLM-induced injury. On day 28, the mice were sacrificed by excessive anesthesia for the collection of lungs and bronchoalveolar lavage fluid (BALF). To generate a rat model of chronic pulmonary fibrosis, SD rats were given SiO_2_ (100 mg/kg) intratracheally. The rats were intragastrically administered with solvent (sham group), or rupatadine at 1, 2 and 4 mg/kg from day 60 to day 90. On day 90, the rats were sacrificed, and a lung was obtained for histological and other examination.

### Lung index

Lung index was determined by lung weight (mg) versus body weight (g) [5].

### Morphological evaluation of lung sections

Histopathological examination was performed as described previously [7]. Lung sections (5-μm-thick) were stained with hematoxylin and eosin (H&E) or Sirius Red and examined with light microscopy. Integrated optical density (IOD) of each section was analyzed by Image-Pro Plus image analysis software. Analysis of mast cells in lung sections was performed as described previously [18].

### Measurement of lung hydroxyproline

Collagen deposition was determined by assaying total hydroxyproline content of the lung according to the revised Reddy GK’s method as described previously [19].

### Morphological evaluation of lung sections

The grades of pulmonary inflammation and fibrosis were analyzed by a professional pathologist, who was blinded for groups. Inflammation was scored individually from 0-5 according to the lesion degree. No fibrosis or inflammation (scored 0), minimal inflammatory changes and alveoli partly enlarged, but no fibrotic masses present (scored 1), mild-to-moderate inflammatory changes without lung structure destruction (scored 2), moderate inflammatory changes with alveolar septal thickening (scored 3), moderate-to-severe inflammatory changes with pneumonitis and fibrosis, destruction of alveolar septa (scored 4), severe inflammatory changes with significant parenchymal destruction (scored 5).

### Preparation of BALF macrophage

Analysis macrophages in BALF was performed as described previously [20].

### Lung functions

Mice were anesthetized with 50 mg/kg i.p. pentobarbital and placed on a flexivent system (ﬂexiVent, SCIREQ Inc., Montreal, Canada). Mice were mechanically ventilated with a tidal volume of 10 ml/kg and 150 breaths/min respiratory rate for evaluating lung functions as described previously [21].

### Acquisition of Microcomputed Tomography Images

Microcomputed tomography (micro-CT) images were generated as described previously [22]. Animals were anesthetized i.p. with pentobarbital (50 mg/kg) to achieve prolonged sedation. The animals were placed in the μCT system (Quantum FX Claiper Inc., CA USA) for imaging. Respiratory gating and cardiac gating were used to stabilize the data. The voxel matrix was 512 × 512 × 512 and the voxel size was 10 × 10 × 10 μm. The exposure time was 17 s, and the reconstruction time was 150 s. Reconstructed images were converted to Hounsfield units (HU) by scaling air to -1000 HU and water to 0 HU. The 3D reconstruction of lung tissue and HU analysis were performed by Quantum Viewer software.

### Cell culture

The murine transformed lung epithelial cells (MLE-12; American Type Culture Collection, Manassas, VA) were cultured in DMEM-F12 supplemented with 2% FCS and 1000 units/mL penicillin and 1000 μg/mL streptomycin. Cells were cultured at 5% CO_2_ in a humidified incubator. To generate senescent cells *in vitro*, MLE-12 cells were exposed to BLM (3 μg/ml), histamine (10 μg/ml), or C-PAF (5 μg/ml) for 96 hours.

### Cell cycle analysis and cell sorting

Cell were harvested and stained with partec CyStain® DNA 1 step (Münster, Germany). The cells were later analyzed by flow cytometry (Partec, Münster, Germany). Cell sorting was performed based on the True Volumetric Absolute Counting technology of the Partec flow cytometer. At least 50,000 stained cells were analyzed using FCS Express software.

### Immunofluorescence

Cells were plated on glass coverslips and subsequently fixed in 4% paraformaldehyde. Coverslips or lung sections were next blocked and permeabilized in 1% bovine serum albumin, 0.1% Triton X-100 in phosphate buffered saline. Specific binding of primary antibodies was detected using corresponding secondary antibodies. The distribution of iNOS and Arg-1 in macrophages and the co-localization of LAMP-1 and LC3 were examined using a TCS SPE fluorescence microscope (Leica, Mannheim, Germany).

### Senescence-associated β-galactosidase staining

Cells were plated on glass coverslips and then detected with senescence-associated β-galactosidase Staining Kit (BioVision, Inc.) following the manufacturer’s instructions.

### Western blotting

Proteins were extracted from lung tissue using a RIPA buffer which contains 1% NP-40 and 1% sodium deoxycholate (Cell Signaling Technology, Danvers, MA). Protein concentrations were determined using a BCA Protein Assay Kit. SDS-PAGE and Western blotting were conducted as described previously [7].

### Statistics

Data are expressed as the mean ± standard error of the mean (SEM). Groups were compared by one-way ANOVA followed by a Tukey-Kramer’s or Dunnett’s multiple comparisons test. Comparisons between two groups were performed by unpaired Student’s t tests. The survival rates were analyzed by the Kaplan-Meier method. A P-value <0.05 was considered to be significant.

## Results

### Rupatadine attenuates BLM-induced pulmonary fibrosis

**Figure 1:**
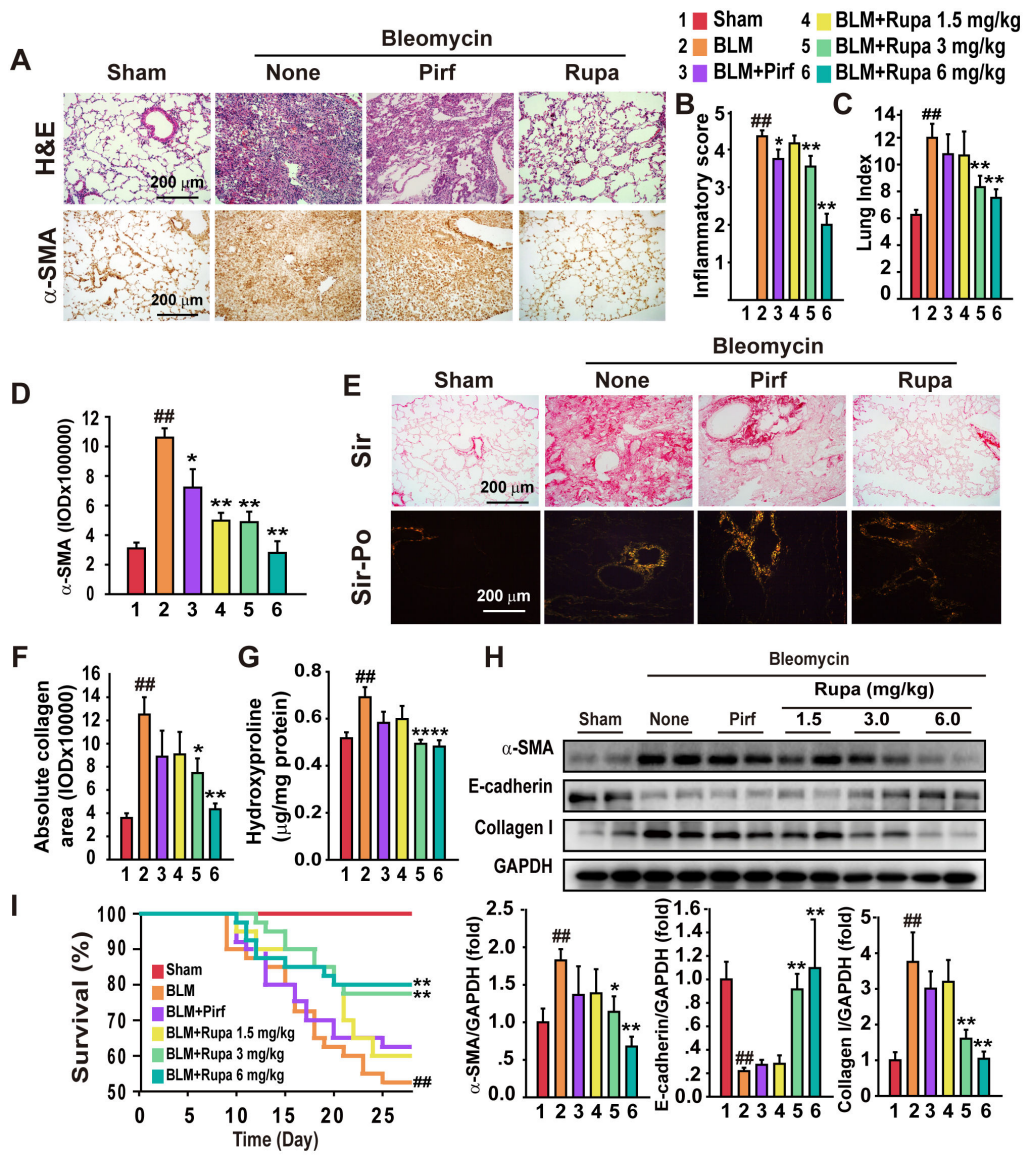
Rupatadine attenuates BLM-induced pulmonary fibrosis. The mice were intragastrically administered with solvent alone (sham group) or rupatadine at 1.5, 3.0, or 6.0 mg/kg per day from day 10 to 28 after BLM administration (5 U/kg). On day 28, mice were sacrificed and a lung was obtained for histological analysis and other studies. (**A**–**D**) Rupatadine attenuated pulmonary fibrosis and inflammation. Representative H&E staining data are shown (A, top), and the expression of α-SMA in fibrotic lungs. The lung sections were stained with an anti-α-SMA antibody for immunohistochemistry analysis (A, bottom). The IOD of each section was analyzed by Image-Pro Plus image analysis software (D) (n=10 per group). Inflammatory score was evaluated by a professional pathologist who was blind to the animal groups (B) (n=15 per group). Rupatadine treatment reduced the lung index in a dose-dependent manner (C) (n=10 per group). (**E**–**G**) Rupatadine decreased collagen deposition in the lungs. The lung tissue sections were stained with Sirius Red (SR) (normal light and polarized light) to indicate the collagen deposition (E and F). Additionally, rupatadine decreased hydroxyproline contents in fibrotic mice (**G**). Scale bar in images = 200 μm. The representative images in A and E were obtained from animals treated with rupatadine of 6.0 mg/kg per day. Data are expressed as the mean ± SEM of 8 mice per group. Rupatadine treatment inhibited the fibrosis-associated molecules in the fibrotic lung tissue (**H**). Western blot analysis was performed on lung lysates and detected the expression of α-SMA, E-cadherin and collagen-I in lung tissue. Data were expressed as folds of the sham group ± SEM of 8 mice per group. Rupatadine decreased animal death in BLM-injured mice (**I**). The cumulative survival rates of mice were analyzed by the Kaplan-Meier method (n=40 per group which were at the start of the experiment). ^#^ *P*<0.05, ^# #^ *P*<0.01 vs. Sham group; ^*^ *P*<0.05, ^**^ *P*<0.01 vs. BLM treated group.

As previously reported, BLM-injured lungs have enhanced infiltration of inflammatory cells and the loss of normal alveolar structure compared with sham-treated mice. The treatment of these mice with rupatadine significantly decreased the infiltration of inflammatory cells, relieved edema and thrombus, and reduced lung structure destruction (Figure 1A top). Rupatadine inhibited, in a dose-dependent manner, α-smooth muscle actin (α-SMA), which is a marker of mesenchymal cells [23], in the BLM-injured lung (Figure 1A bottom and D). Additionally, rupatadine markedly decreased the BLM-enhanced inflammatory scores (Figure 1B) and lung index (Figure 1C) compared to BLM-treated mice. Sirius Red analysis showed a significant collagen deposition in fibrotic lung tissue, particularly around the bronchi. Rupatadine also reduced collagen deposition (Figure 1E and F) and hydroxyproline content (Figure 1G) in a dose-dependent manner. The BLM-injured lung showed a significant increase in the expression of α-SMA, and this increase could be blocked by the treatment of these mice with rupatadine (Figure 1H). However, the expression of E-cadherin, a biomarker of epithelial tissues [24], was inhibited in fibrotic lungs, and this action was blocked by the treatment of mice with rupatadine. The anti-fibrotic effect of rupatadine decreased the expression of collagen type I (Figure 1H). Thus, treatment of these mice with 3 or 6 mg/kg per day of rupatadine decreased animal death (Figure 1I). However, pirfenidone, an approved drug for pulmonary fibrosis [25], showed neither therapeutic efficacy for pulmonary fibrosis nor decrease in animal death, although it diminished the inflammatory response (Figure 1).

The anti-fibrotic role of rupatadine was compared with the H1 receptor antagonist loratadine, PAF receptor antagonist CV-3988, or the drug combination of loratadine plus CV-3988 in BLM-induced pulmonary fibrosis. We found that rupatadine had the best therapeutic profile in terms of anti-inflammation and anti-collagen deposition (Figure S1A). Moreover, rupatadine or the drug combination but not loratadine or CV-3988 alone significantly reduced the hydroxyproline contents in fibrotic lung tissue (Figure S1B). Thus, only these two regimens increased animal survival (Figure S1C). Rupatadine significantly inhibited the recruitment of inflammatory cells in BALF, but H1 or PAF antagonists alone showed a weak inhibition of inflammation (Figure S1D).

### Rupatadine improves lung structure and functions in fibrotic mice

**Figure 2:**
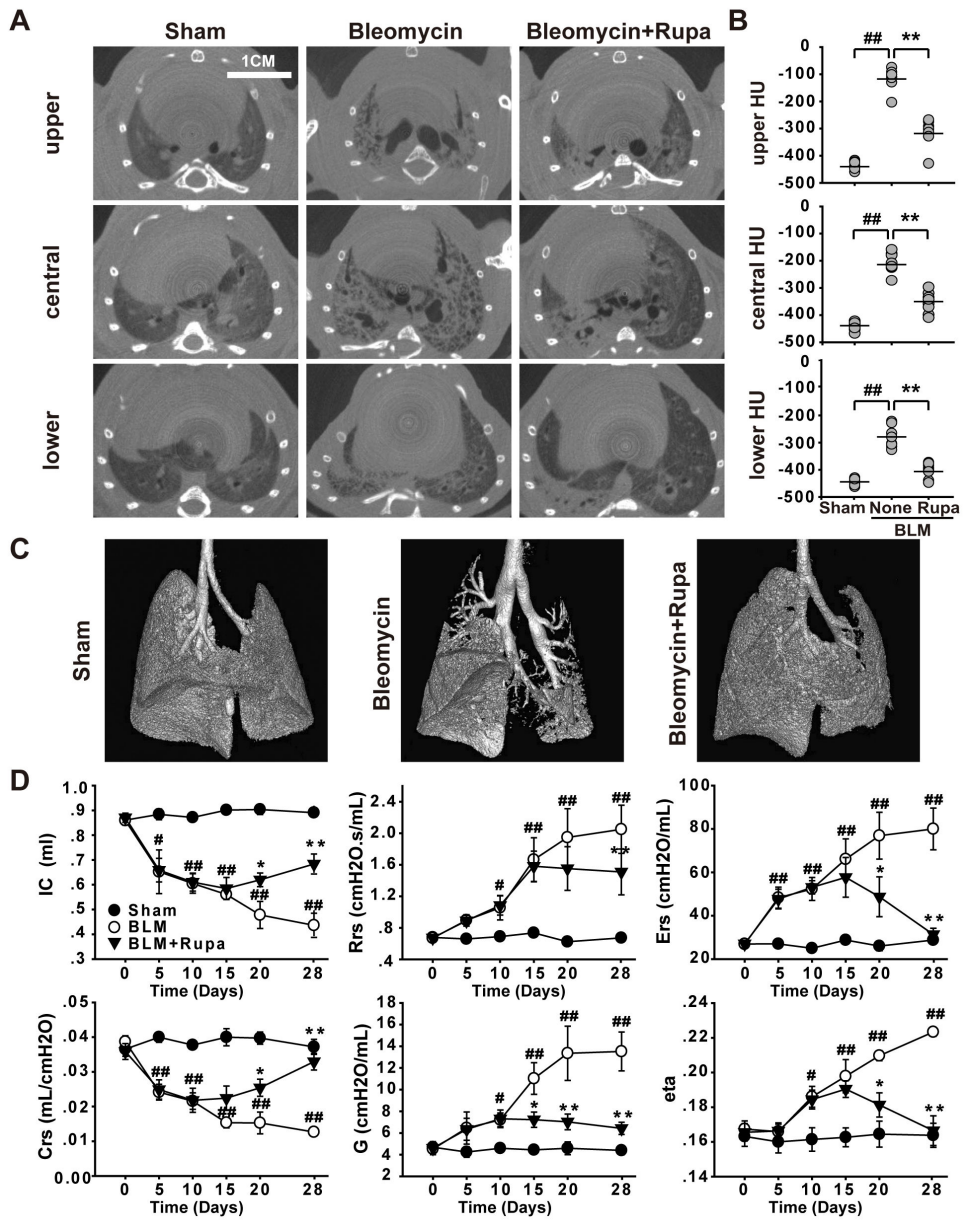
Rupatadine reduces enhanced lung density and improves lung functions in fibrotic mice. (**A**–**C**) Rupatadine treatment reduced the BLM-induced lung density shown by micro-CT. Representative micro-CT of main pulmonary lesions of Sham- (A, left), BLM instilled- (A, middle) and rupatadine-treated mice (6.0 mg/kg per day) (A, right) were shown at different slices. Quantification of lung parenchyma density was measured in upper, central and lower pulmonary regions excluding the hilum and bronchi. Scale bar in images = 1 cm. The data are expressed as the mean Hounsfield units (HU) ± SEM of 8 mice per group (B). Rupatadine (6.0 mg/kg per day) reduced parenchymal loss in the fibrotic mice (C). (**D**) Rupatadine (6.0 mg/kg per day) improved lung functions in the fibrotic mice. Mice were anesthetized with 50 mg/kg i.p. pentobarbital and placed on the flexivent system at the indicated times after bleomycin administration. Mice were mechanically ventilated with a tidal volume of 10 ml/kg and a respiratory rate of 150 breaths/min. The parameters of lung function were calculated by measuring total lung capacity, Snapshot, Quickprime-3, and pressure-volume loops. All perturbations were performed until three acceptable measurements with a coefficient of determination (COD) ≥ 0.9 were recorded for each individual subject. The data are expressed as the mean ± SEM of 6 mice per group. ^#^ *P*<0.05, ^# #^ *P*<0.01 vs. Sham group; ^*^ *P*<0.05, ^**^ *P*<0.01 vs. BLM treated group.

Micro-CT was utilized to observe lung consolidation. We found severe consolidation and reticular shadow with enhanced density in the different slices of both lobes in un-treated fibrotic mice; the treatment of mice with 6.0 mg/kg of rupatadine promoted the absorption of the lesions and decreased the density of lungs (Figure 2A and B). We also found that rupatadine recovered a volume of functional lung tissue after BLM-induced injury, which was shown by three dimensional reconstruction of CT scans (Figure 2C). Because rupatadine ameliorated lung structure in the fibrotic mice, we examined if rupatadine improved lung functions. We found that rupatadine significantly increased inspiratory capacity (IC); reversed the high dynamic resistance (Rrs), dynamic elasticity (Ers), and low dynamic compliance (Crs); and decreased the tissue damping (G) and tissue hysteresivity (eta) (Figure 2D) in the fibrotic mice. These data indicate that rupatadine can improve the fibrotic structure and lung functions in fibrotic animals.

### Rupatadine diminishes inflammatory responses

**Figure 3:**
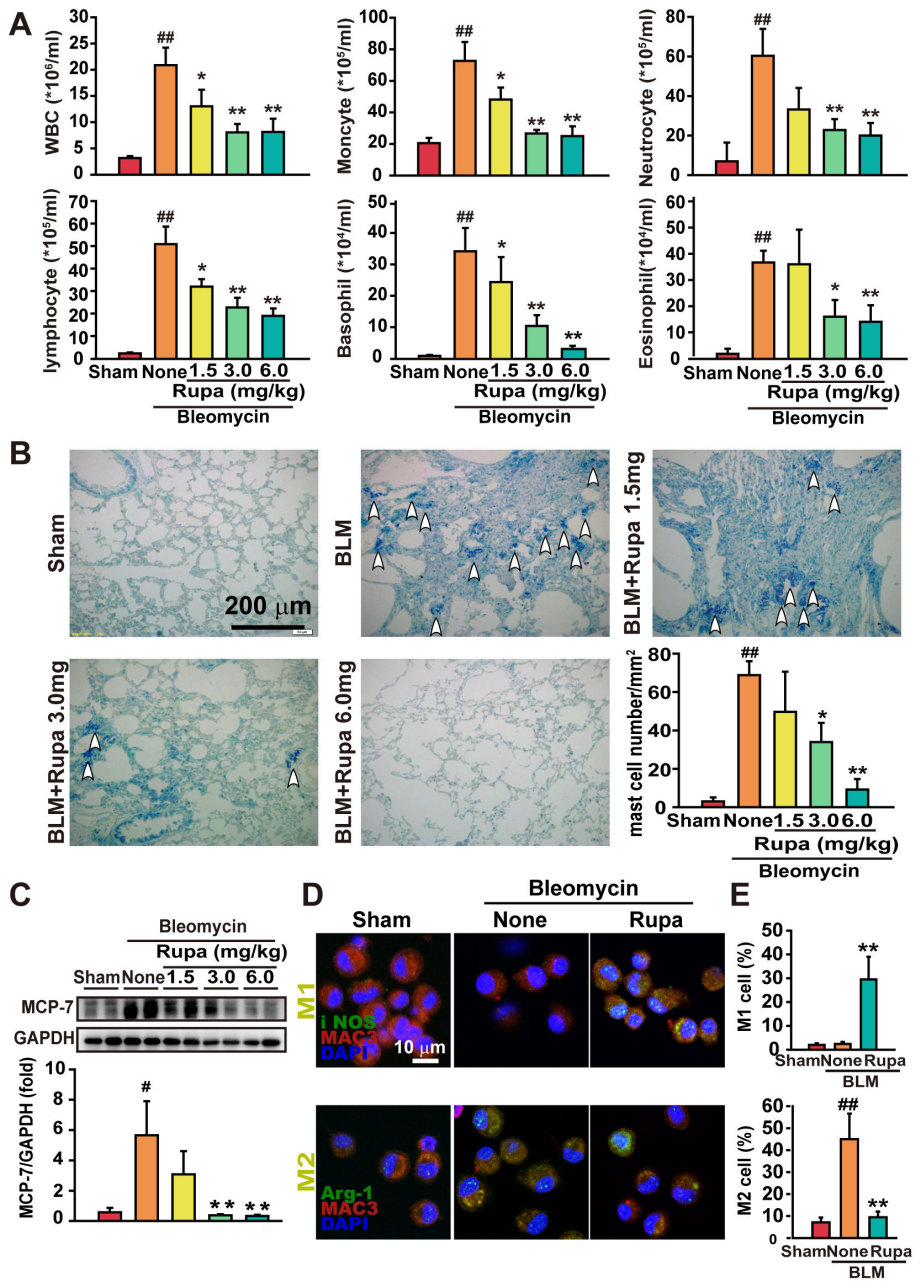
Rupatadine promotes the resolution of inflammatory responses. (**A**) BALF was collected on day 28 after BLM administration, and the counts of total WBC and classified cells were evaluated by differential counting. The data are expressed as the mean ± SEM of 8 mice per group. (*B* and *C*) Rupatadine decreased the population of lung-infiltrating mast cells and stabilized their activity in the fibrotic lung tissue. The density of mast cells in examined lung sections was detected by staining with 0.05% (w/v) toluidine blue (shown by arrows). The data are representative images of 3 assays with similar results. The mast cells (shown by arrow) were counted in 15 fields at 400x of each mouse. The data are representative images and are summarized as the mean ± SEM of 8 mice per group. Scale bar in images = 200 μm(B). The expression of MCP-7, a specific protease secreted by mast cell degranulation, was detected by Western blotting (C). The data are representative immune blots and summarized as the mean ± SEM of 8 mice per group. (**D** and **E**) Rupatadine (6.0 mg/kg per day) regulated the infiltration and polarization of macrophages in BALF in the fibrotic mice. Images of BALF cells on glass coverslips were acquired by confocal microscopy for MAC-3 (red) iNOS (green in top) and Arg-1 (green in bottom). The data are representative images of 3 assays. Scale bar in images = 10 μm(D). M1 and M2 cells on the sections were analyzed by Image-Pro Plus software and expressed as the mean ± SEM of 6 mice per group (E). ^#^ *P*<0.05, ^# #^ *P*<0.01 vs. Sham group; ^*^ *P*<0.05, ^**^ *P*<0.01 vs. BLM treated group.

To determine the effects of rupatadine on inflammatory responses, lung-infiltrating inflammatory cells and their subtypes in BALF were analyzed. We found that WBC counts, as well as monocytes, basophils, neutrophils and eosinophils, were significantly elevated in fibrotic mice compared with sham mice. However, the mice treated with rupatadine showed a dose-dependent reduction in the infiltration of inflammatory cells (Figure 3A). Moreover, rupatadine reduced elevated IL-2 levels but did not change the content of IL-12 and IFN-γ in BALF (Figure S2A). However, rupatadine markedly decreased the levels of the pro-fibrotic cytokines IL-4, IL-10, IL-17 and TGF-β in BALF. Notably, the contents of PAF and histamine were significantly increased in BALF from BLM treated mice, but rupatadine decreased the levels of these allergic mediators (Figure S2A).

Mast cells are the most crucial target cells of rupatadine in the treatment of allergic diseases [26]. We found that rupatadine decreased mast cell infiltration in the fibrotic lungs, as detected by Toluidine Blue stain (Figure 3B). Consistently, rupatadine treatment caused a dose-dependent decrease in the expression of mast cell protease-7 (MCP-7), which is specifically expressed in mast cell secretory granules and may serve as a highly specific marker in the analysis of mast cell heterogeneity, differentiation, and function (Figure 3C) [27].

M1 or M2 macrophages play opposite roles in the pathogenesis of pulmonary fibrosis, and the balance of M1/M2 determines the properties of inflammatory responses [28]. We thus examined BALF cells with confocal microscopy for detecting macrophage marker MAC-3, M1 marker iNOS, and M2 marker Arg-1 [29]. We found that the BALF cells from sham mice showed an iNOS ^low^Arg-1^low^ phenotype. The BALF cells from the BLM-treated mice displayed an iNOS ^low^Arg-1^high^ phenotype, indicating M2 polarization; the BALF cells from the rupatadine-treated mice exhibited an iNOS ^high^Arg-1^low^ phenotype, indicating M1 polarization (Figure 3D and E).

We examined the expression of inflammatory cytokines in lung tissues. Rupatadine treatment significantly decreased the expressions of IL-1, TNF-α, IL-6, IL-10, IL-17 and TGF-β in a dose-dependent manner (Figure S2B), suggesting that rupatadine could ameliorate the BLM-stimulated inflammatory response. Although the expression of the Th1 transcription factor T-bet was not changed, the expression of Th2 transcription factor GATA-3 was significantly increased in the fibrotic lungs (Figure S2C). Rupatadine markedly reduced GATA-3 expression but increased T-bet expression (Figure S2C). In addition, rupatadine inhibited the expression and activity of NF-κB, which was increased in model group. Transcription factor STAT3 is associated with chronic inflammatory diseases and the transcription products of STAT3, such as IL-6, IL-10, and IL-17, are involved in the pathogenesis of pulmonary fibrosis [30]. We found that BLM did not affect the expression of STAT3 but increased the ratio of pSTAT3 to STAT3. Rupatadine suppressed the phosphorylation of STAT3 in fibrotic lung tissue (Figure S2C).

### Rupatadine attenuates cellular senescence

**Figure 4:**
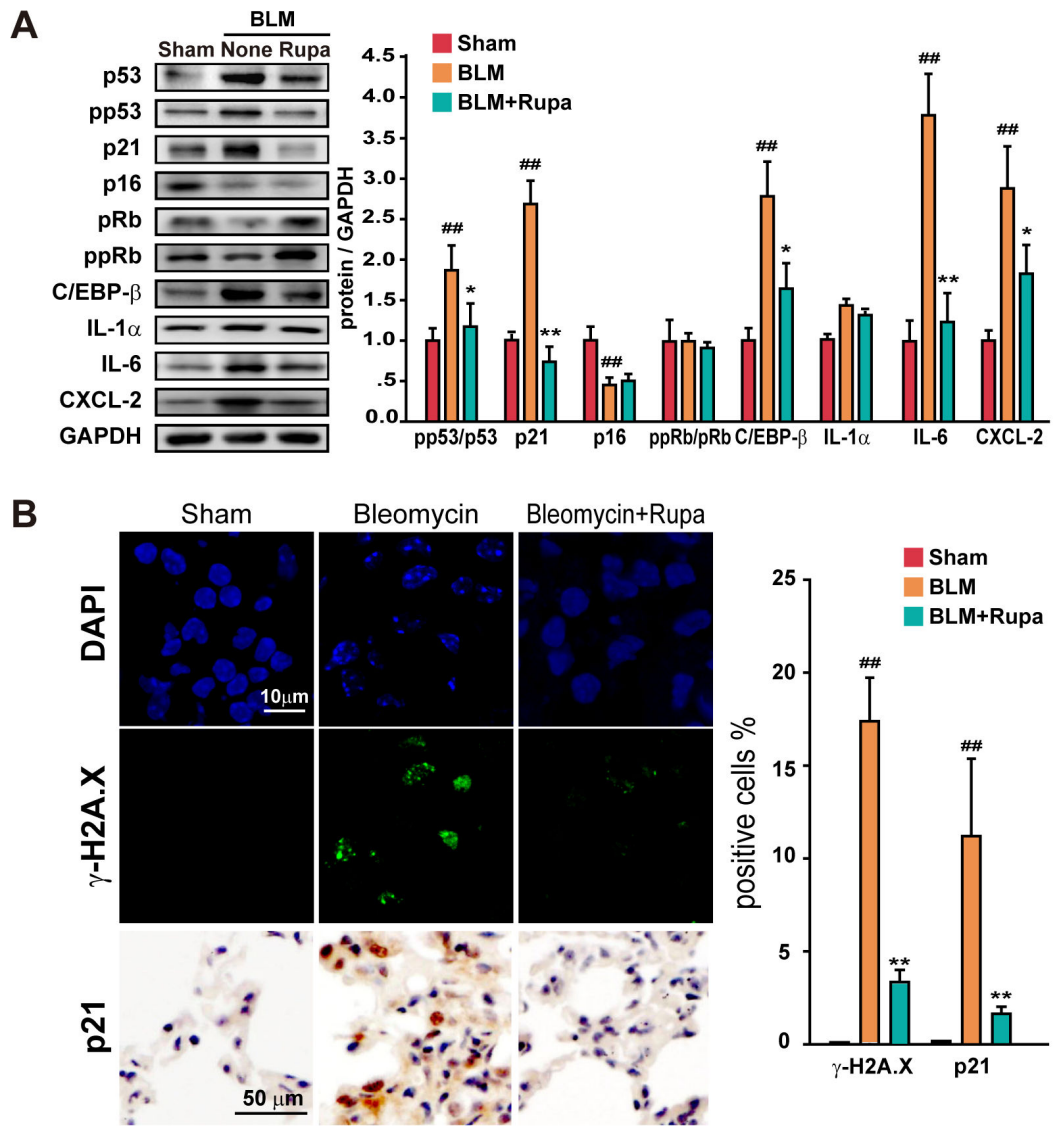
Rupatadine attenuates cellular senescence in fibrotic mice. (**A**) Rupatadine (6.0 mg/kg per day) attenuated the expression or activity of senescence-related molecules in fibrotic lung tissue. Lung tissue extract was prepared for western blotting. The data are representative immune blots and expressed as the mean ± SEM of 8 mice per group. (**B**) Rupatadine (6 mg/kg) reduced the number of senescent cell in fibrotic mouse lung tissue. Representative images of lung sections were acquired by confocal microscopy for DAPI (blue)/ γ-H2A.X (green) or immunohistochemistry for p21. Scale bar in images = 10 μm or 50 μm. The data are expressed as the mean ± SEM of 5 mice per group. ^#^ *P*<0.05, ^# #^ *P*<0.01 vs. Sham group; ^*^ *P*<0.05, ^**^ *P*<0.01 vs. BLM treated group.

Lung epithelial cell injury is the origin of pulmonary fibrosis, but how the injured epithelial cells participate in the fibrotic process is controversial. Recent studies indicate that stress- or DNA damage-induced premature senescence in these cells may play a critical role in the pathogenesis of pulmonary fibrosis [31]. Thus, we suspected that rupatadine could regulate senescence to ameliorate pulmonary fibrosis in lung epithelial cells. Indeed, BLM-induced fibrotic lung tissue showed a p53/21-dependent induction of premature senescence (Figure 4A). Treatment of these mice with rupatadine decreased the expression or activity of p53 and p21 in BLM-injured lung tissue with no influence on the expression of p16 and the ratio of p-pRb and pRb (Figure 4A). C/EBP-β is a positive modulator of cellular senescence, which activates the transcription of inflammatory cytokines, such as IL-1α, IL-6, IL-8, and other Senescence-Associated Secretory Phenotype (SASP) factors [32]. Rupatadine attenuated the enhanced expression of C/EBP-β, IL-6, and CXCL-2 (a mouse ortholog of human IL-8) but not IL-1α in the fibrotic lung tissue (Figure 4A). We also found that there were many senescent cells in the lung tissue of fibrotic mice, and these cells showed senescence-associated heterochromatin foci and γ-H2A.X foci dual positive (Figure 4B) which occurs spontaneously with senescent response [32]. The distribution of p21 in the lung tissues also indicated a senescence response after bleomycin injury (Figure 4B). Rupatadine markedly decreased the number of DNA damaged senescent cells and the expression of p21 in fibrotic mouse lungs (Figure 4B).

**Figure 5:**
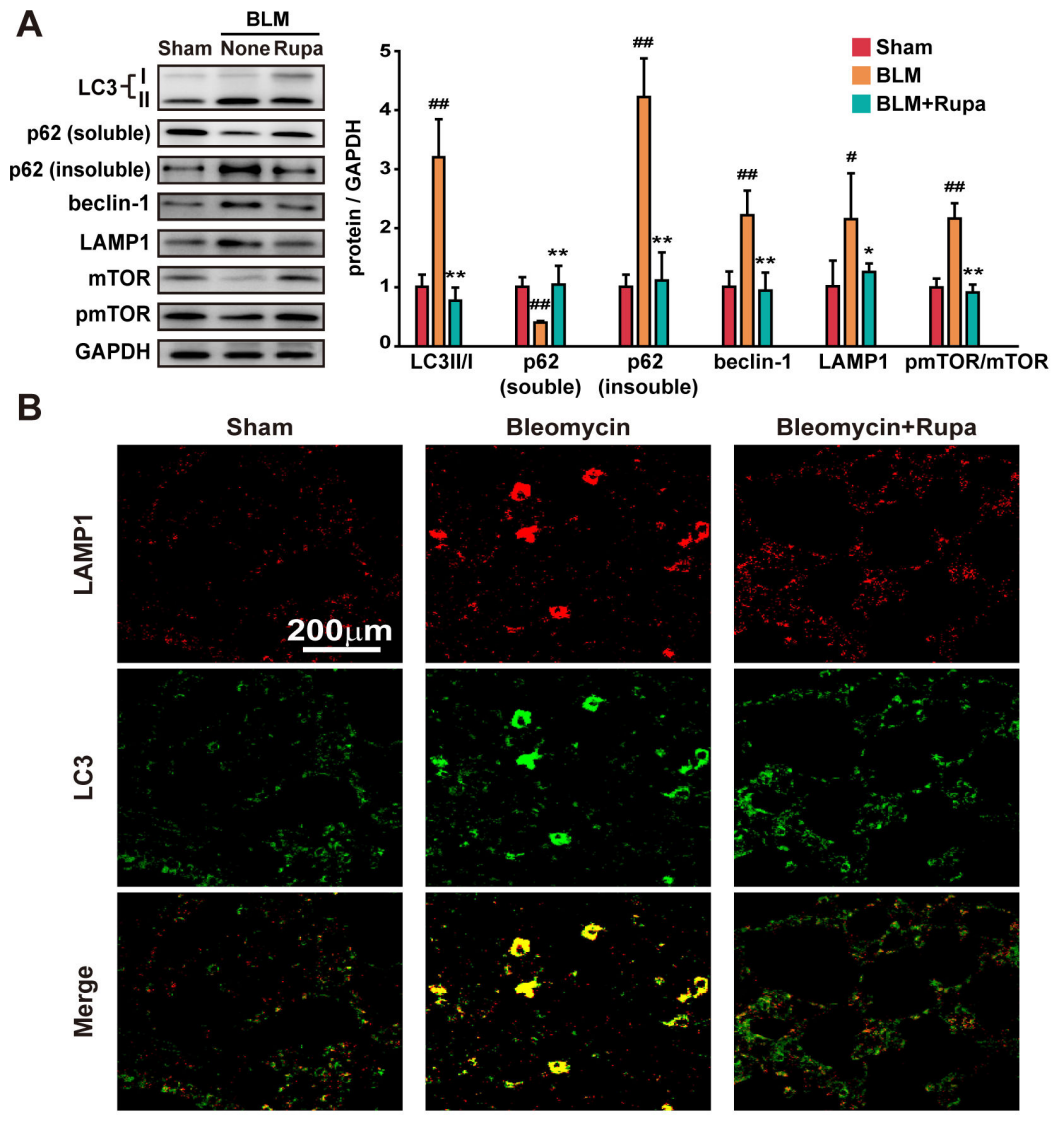
Rupatadine maintains autophagic flux in fibrotic lung tissue. (**A**) Rupatadine (6.0 mg/kg per day) regulated the expression or activity of autophagy-related molecules in the fibrotic lung tissue. Lung tissue extract was prepared for western blotting. The data are representative immune blots and expressed as the mean ± SEM of 6 mice per group. (**B**) Rupatadine (6.0 mg/kg per day) maintained the autophagic flux in the fibrotic lung tissue. Representative images of lung sections were acquired by confocal microscopy for LC3-II (green)/ LAMP1 (red). Scale bar in images = 200 μm. The data are representative images of 3 assays with identical results. ^#^ *P*<0.05, ^# #^ *P*<0.01 vs. Sham group; ^*^ *P*<0.05, ^**^ *P*<0.01 vs. BLM treated group.

The activation of autophagy is an indicator for senescence induction [33]. BLM-injured lung tissue showed increases in the conversion of LC3I/II and the expression or activity of autophagy-related signal molecules baclin-1 and pmTOR (Figure 5A). The fibrotic lung tissue displayed a decrease of “cargo protein” p62 in a soluble form but an increase in an insoluble form, indicating that p62 aggregates accumulated and that autophagic flux was impaired in the fibrotic lung tissue [34]. Rupatadine increased the phosphorylation of mTOR but did not change the expression of beclin1. However, rupatadine decreased the expression of insoluble p62 (Figure 5A) and reduced the co-localization of LAMP-1 and LC3 in the fibrotic lung tissue (Figure 5B). These data suggest that rupatadine relieved the impaired autophagy flux in the fibrotic lung tissue.

### Rupatadine antagonizes silica-induced chronic pulmonary fibrosis

To investigate if rupatadine antagonized chronic pulmonary fibrosis, a rat model of silica-induced pulmonary fibrosis was generated, as described previously [7]. The rats were treated with rupatadine for 30 days. We found that rupatadine diminished silica-induced inflammation and decreased the number of silicon nodes and collagen deposition (Figure S3A). Thus, rupatadine improved silica-damaged lung functions, as indicated by reducing silica-enhanced airway dynamic resistance, dynamic elasticity, and blocking the silica-induced low dynamic lung compliance (Figure S3B). Additionally, rupatadine decreased TGF-β level in BALF (Figure S3C), hydroxyproline content (Figure S3D) and α-SMA expression in a dose-dependent manner (Figure S3E). These data indicate that rupatadine can antagonize the silica-induced chronic pulmonary fibrosis.

### Rupatadine inhibits PAF-mediated p53-dependent senescence

**Figure 6:**
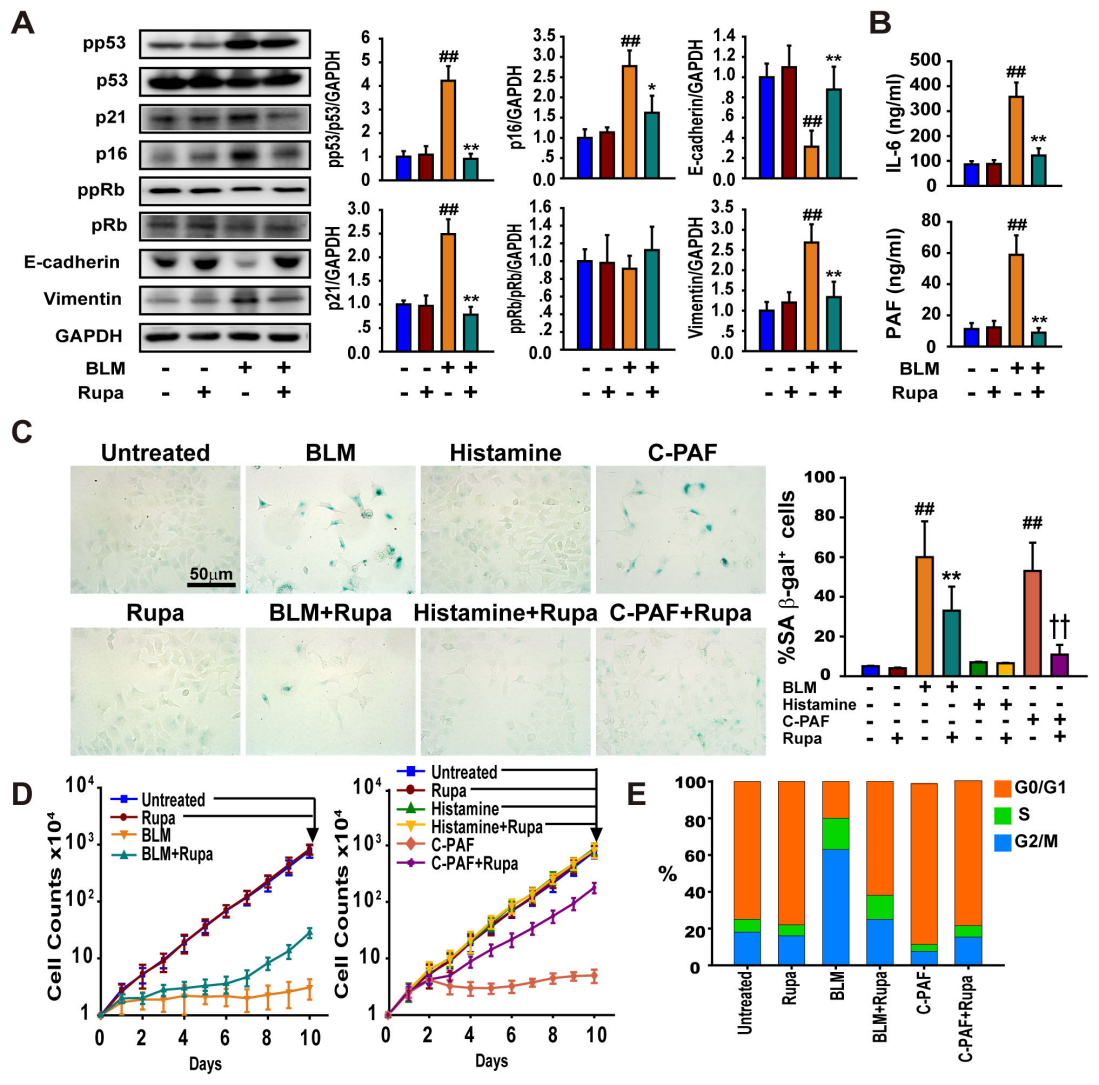
Rupatadine inhibits BLM- and PAF-induced epithelial cellular senescence. (**A**) Rupatadine inhibited the expression or phosphorylation of the BLM-induced senescence-related molecules in MLE-12 cells. The data are representative immune blots and expressed as the mean ± SEM of four independent assays. (**B**) Rupatadine inhibited the secretion of IL-6 and PAF from the BLM- and PAF-induced senescent MLE-12 cells. The content of IL-6 and PAF in supernatant solutions was detected by ELISA kits. The data are expressed as the mean ± SEM of four independent assays with triplicates. (**C**) Rupatadine inhibited the expression of SA β-gal induced by BLM and C-PAF. MLE-12 cells were planted on coverglass-bottom dishes and treated with BLM (3 μg/ml), rupatadine (25 μg/ml), histamine (10 μg/ml), or C-PAF (5 μg/ml) for 96 hours. MLE-12 cells were stained by a senescence kit and examined for SA β-gal activity (blue). Scale bar in images = 50 μm. (**D** and **E**) Recovery from BLM and C-PAF induced growth arrest by rupatadine. MLE-12 cells were cultured in the presence or absence of BLM (3 μg/ml), rupatadine (25 μg/ml), histamine (10 μg/ml), and C-PAF (5 μg/ml) for 10 days. Population doubling (D) and representative DNA profile for the treated cells at day 10 were examined by flow cytometry (E). ^#^ *P*<0.05, ^# #^ *P*<0.01 vs. Untreated group; ^*^ *P*<0.05, ^**^ *P*<0.01 vs. BLM treated group; † † *P*<0.01 vs. C-PAF group.

The regulatory role of rupatadine in BLM-induced senescence was studied in cultured lung epithelia cells. We found that rupatadine significantly inhibited BLM-induced cellular senescence (Figure 6A). Interestingly, rupatadine reduced the expression of the mesenchymal marker vimentin and enhanced the expression of E-cadherin (Figure 6A). Not only the secretion of IL-6 but also PAF was significantly increased in the BLM-induced senescent cells, illustrating that PAF and IL-6 are the members of SASP and might play a similar regulatory role in the senescent process (Figure 6B). Indeed, after MLE-12 cells were treated with BLM, histamine or C-PAF for 96 h, BLM, and C-PAF but not histamine treated cells displayed a typical phenotype of senescence as indicated by an increase in SA β-gal activity (Figure 6C) which is a biomarker of senescent cells [32] and distinct morphology (data not shown). Importantly, rupatadine could inhibit the BLM- and C-PAF-induced growth arrest of MLE-12 cells and promote the transition of senescent cells into normal growth status (Figure 6D). Consistently, the BLM-treated cells were growth-arrested at the G2/M phase of the cell cycle, but the C-PAF-treated cells were growth-arrested at the G0/G1 phase (Figure 6E). Rupatadine not only induced the entry of BML-treated cells to the growth phase of G0/G1 but also made the C-PAF-treated cells enter the G2/M growth phase (Figure 6E).

**Figure 7:**
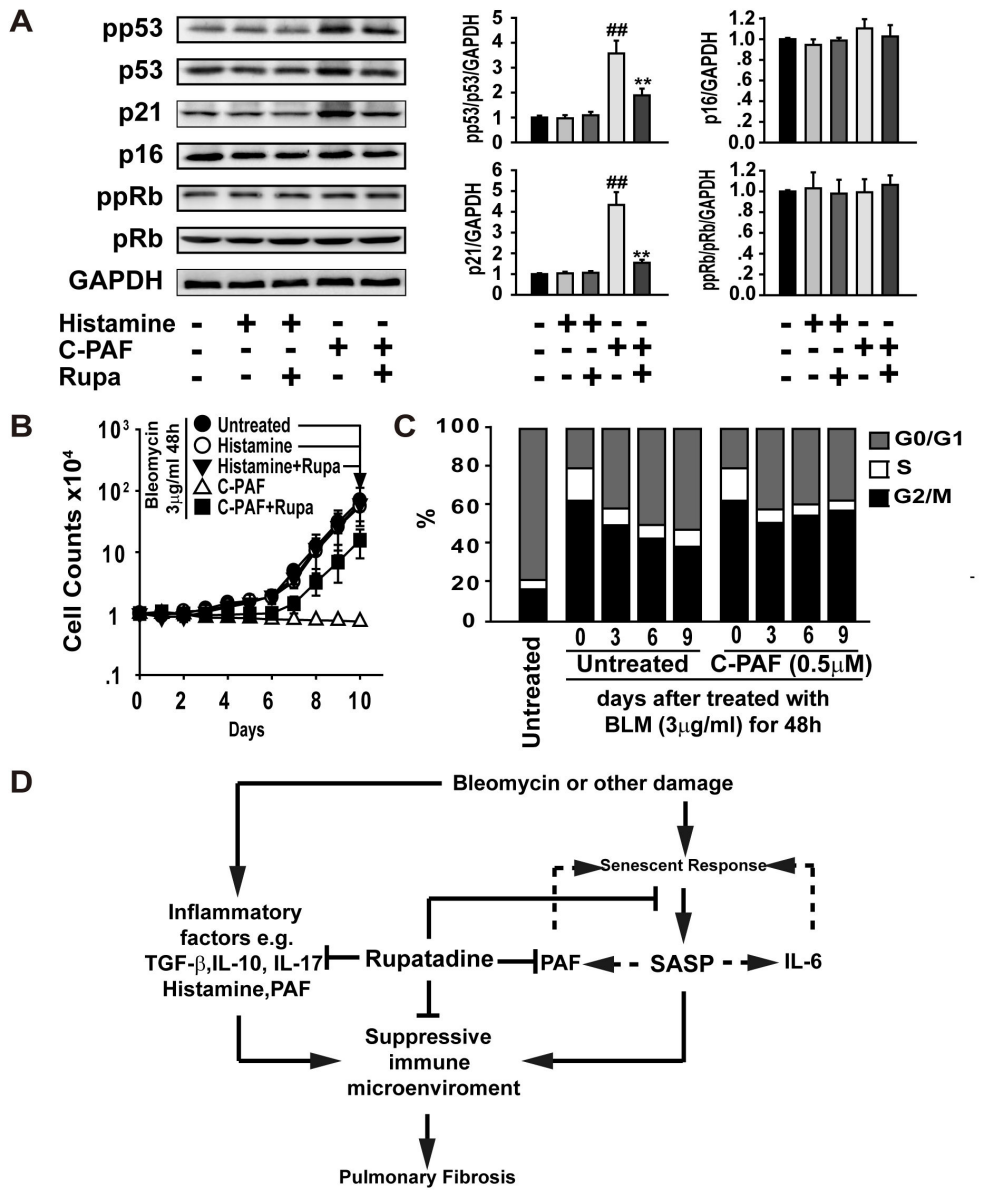
PAF induces and sustains p53-dependent senescence. (**A**) C-PAF (5 μg/ml), but not histamine (10 μg/ml), induces the expression or phosphorylation of the senescent-related signal molecules in lung epithelial cells. The data are representative immune blots and are expressed as the mean ± SEM of four independent assays. (**B**) The lower concentration of C-PAF but not histamine induces the growth arrest of lung epithelial cells. MLE cells were treated with BLM (3 μg/ml) for 48 h followed by the withdrawal of BLM and further incubation for 10 days with or without histamine (1 μg/ml), C-PAF (0.5 μg/ml) or rupatadine (25 μg/ml). (**C**) C-PAF sustained the cell cycle arrest of senescent cells. Bar graphs show the percentage of MLE-12 cells in G0/G1, S and G2/M phase. (**D**) Schematic diagram illustrating the mechanism of rupatadine in the treatment of pulmonary fibrosis. The BLM-induced acute inflammation may convert to chronic inflammation leading to pulmonary fibrosis progression via 1) Immunosuppressive cells and soluble factors that interfere with the resolution of inflammation. 2) The injured lung tissue expresses the SASP to secret soluble factors that sustain senescence and inflammation. Rupatadine can antagonize the activation of inflammatory cells, such as mast cells, and partly inhibits the BLM- and PAF-mediated senescence response both *in vivo* and *in vitro*.

Consistently with these findings, C-PAF but not histamine activated the p53/p21 signaling, but rupatadine completely blocked the C-PAF-induced activation of the p53/p21 (Figure 7A). Thus, we investigated if C-PAF could sustain the p53/p21-dependent senescence response similar to the activity of IL-6 observed previously [35]. MLE-12 cells were incubated with BLM (3 μg/ml) for 48 h, and BLM was withdrawn from the media. The cells were stimulated with a lower concentration C-PAF or histamine, and these concentrations of stimulators could not induce senescence in MLE-12 cells (data not shown). We found that BLM-induced senescent cells regained their growth ability after BLM withdrawal (Figure 7B). Notably, C-PAF but not histamine could maintain senescence response after BLM withdrawal. Indeed, MLE-12 cells were gradually recovered from G2/M growth arrest to the normal cell cycle, but C-PAF inhibited this change (Figure 7C).

## Discussion

Pulmonary fibrosis is driven by chronic inflammation by the heterogenic properties of pathogens, immune cells, and soluble factors [1]. However, chronic inflammation in fibrotic lung tissue is characterized by a suppressive property of the immune microenvironment [5]. The activated resident cells and the lung-infiltrating immunosuppressive cells, including Th2, Th9, Th17, Treg, mast, and M2 cells, produce a large amount of immunosuppressive soluble factors [5,7,12,28,36]. These factors can interfere with inflammation resolution, promote tissue repair processes, and induce injury in epithelial cells, leading to extracellular matrix deposition. Thus, unresolved inflammation and abnormal tissue repair promotes the progression of pulmonary fibrosis. Interestingly, recent work indicates that allergic diseases, such as asthma, are more than just Th2-diseases [37]. A heterogenic inflammatory profile similar to fibrotic tissue can be attributed to the pathogenesis of allergic diseases [9]. Moreover, many soluble factors released from these immune cells participate in the development of allergic diseases and pulmonary fibrosis [12–15]. For instance, the allergic molecule histamine is increased in the BALF of pulmonary fibrosis hamsters [38], which induces fibroblast proliferation and migration *in vitro* [13]. Thus, an H1 receptor antagonist modulates the imbalance of Th1/Th2 cytokines and the inflammatory response [39], while PAF receptor antagonist WEB 2086 ameliorates BLM-induced pulmonary fibrosis [40]. Thus, the observations that similar immune cells and soluble factors are implicated in the pathogenesis of pulmonary fibrosis and allergy conditions provide a rationale for using the antiallergic drug rupatadine to target H1 or PAF signaling for treatment of pulmonary fibrosis.

In this study, we find that the treatment of fibrotic animals with rupatadine decreases populations of lung-infiltrating inflammatory cells, including monocytes, basophils, neutrophils, eosinophils, mast cells and macrophages. Consistent with these findings, rupatadine decreases the expression of inflammatory cytokines, inhibits mast cell activation, and regulates the balance of M1/M2 macrophages in BLM-injured lungs. Rupatadine treatment also reverses the immunosuppressive environment in fibrotic lungs. This result is similar to that obtained in our previous work in which targeting TLR2 reversed established pulmonary fibrosis through the reversal of lung injury-induced immunosuppressive environment [5]. Indeed, a previous study indicated that rupatadine can inhibit the secretion of allergic and inflammatory mediators, such as histamine, PAF, TNF-α, IL-6, IL-8, IL-10, and IL-13, from human mast cells and other immune cells [26]. Rupatadine has been an excellent option for the treatment of allergic diseases [16]. Our current studies offer evidence to indicate that rupatadine is a potential option for the treatment of pulmonary fibrosis.

Cellular senescence is an adaptive response that can be induced by many types of stimuli, including oncogene activation, oxidative stress, and DNA damage [41]. Although premature senescence has been recognized as an important mechanism for many chronic inflammatory diseases [41], the role of senescence in fibrotic diseases is controversial. A recent study reported that senescent satellite cells limit the development of liver fibrosis [42]. In contrast, it was evident that senescent epithelial cells exacerbate pulmonary inflammation [43]. BLM can act as a DNA damage reagent and cause cellular senescence in alveolar epithelial A549 cells [44]. In the current study, we found that BLM induces a premature senescence in MLE-12 cells, as characterized by growth arrest, distinct cell morphology, increased SA-β-gal activity, and the expression of senescence-associated molecular signals. Importantly, our studies indicate that the senescence response is induced in fibrotic lung tissue; the treatment of fibrotic mice with rupatadine protects against the progression of pulmonary fibrosis and ceases the establishment of premature senescence in fibrotic lung tissue.

Senescent cells have deleterious effects on fibrotic lung tissue, due to the high level of adverse SASP factors released from these cells, including cytokines, growth factors, proteases, and other factors [34]. IL-1α, IL-6, and IL-8 are the most important cytokines of the SASP factors, which can initiate and maintain the cellular senescence and results in more factors being released from these senescent cells [45]. We found that rupatadine inhibits the expression of IL-6 and CXCL-2 in the BLM- or PAF-induced senescent MLE-12 cells. This property of rupatadine has also been linked with the inhibition of degranulation of allergic cells induced by immunological and non-immunological stimuli. Indeed, the growth arrest of MLE-12 cells induced by BLM was partly reversed after rupatadine treatment, suggesting that BLM-induced senescence is reversible. PAF is a potent phospholipid activator that mediates many leukocyte functions including platelet aggregation and degranulation, inflammation, and anaphylaxis. Our studies demonstrate that PAF is a SASP factor and can induce a senescence response directly *in vitro* (Figure 7D).

Autophagy is an essential developmental program that maintains tissue homeostasis [46], and recent work indicates that autophagy cross-talks with senescence [47]. Generally speaking, senescence induction activates autophagy whereas activated autophagy is an indicator for senescence induction [33]. Conversely, impaired autophagy may leave inflammatory substances, such as pathogens, unfolded proteins, and damaged cells and debris in the injured tissue, which may result in oxidative stress and DNA damage to trigger cellular senescence. Indeed, our recent studies demonstrate that the impaired autophagy may play a critical role in the pathogenesis of pulmonary fibrosis [7,48,49]. A moderate activation of autophagy can attenuate the development of pulmonary fibrosis by clearing the chronic inflammatory substances and directly degrading collagen in the fibrotic tissue. In this study, rupatadine treatment restored autophagy flux and reduced p62 aggregates, which is associated with a decreased senescence response.

Rupatadine is a small molecular drug with good safety profiles and has been approved for use in many countries [50]. The maximum dosage of rupatadine used in our studies has been demonstrated to be within the safety margin in humans [51]. Moreover, the therapeutic administration of rupatadine protects against pulmonary fibrosis, indicating that rupatadine may antagonize established pulmonary fibrosis. Finally, rupatadine shows not only an improved anti-fibrotic efficacy compared to a histamine H1 or PAF receptor antagonist alone, but it also has a better therapeutic effect on pulmonary fibrosis than the combination regimen of H1 and PAF receptor antagonists. Moreover, rupatadine is more effective, at least in our study, than pirfenidone, an approved drug for the treatment of pulmonary fibrosis [25].

In summary, our studies may satisfy an unmet need in the development of a therapeutic agent for the prevention and treatment of fibroproliferative pulmonary diseases. Considering that rupatadine is a safely prescribed drug, our studies shed light on the chronic lung diseases that are resistant to the current therapeutics. Additionally, the treatment of chronic allergy patients with rupatadine may reduce the fibrotic complication associated with allergy diseases. Beside the excellent anti-inflammatory effect, rupatadine could attenuate pulmonary fibrosis through its anti-senescent role. However, there still may be other mechanisms during the course of therapy. In addition, we were also curious whether rupatadine would be effective on other tissue fibrosis.

## Supporting Information

Figure S1 **Anti-fibrotic effect of rupatadine is superior to H1 antagonist loratadine and PAF antagonist CV-3988.** After intratracheally administered BLM (5 U/kg), the mice were intragastrically administered solvent only (Sham group), rupatadine (3.0 mg/kg), loratadine (3.0 mg/kg), CV-3988 (3.0 mg/kg), or 3.0 mg/kg loratadine plus 3.0 mg/kg CV-3988 (Lora + CV-3988) from day 10 to 28. On day 28, the mice were sacrificed and a lung was obtained for histological analysis and other examination. (**A**) Rupatadine treatment had the best anti-inflammation and anti-collagen deposition effects after BLM-injured. Histological examination was performed by hemotoxylin–eosin (H&E) staining (**A**, top) and Sirius Red (SR) staining (**A**, bottom). Scale bar in images = 200 μm. (**B**) Rupatadine and drug combination regimen reduced the hydroxyproline contents in fibrotic mice lung tissue. The data are expressed as the mean ± SEM of 10 mice per group. (**C**) Rupatadine and drug combination regimen significantly elevated survival rate of BLM-injured mice (n=40 per group which were at the start of the experiment). (**D**) BALF was collected on day 28 and classes of leukocytes quantified by differential counting. Rupatadine significantly inhibited the recruitment of inflammatory cells. The data are expressed as the mean ± SEM of 8 mice per group. ^#^ *P*<0.05, ^##^ *P*<0.01 vs. Sham group; ^*^ *P*<0.05, ^**^ *P*<0.01 vs. BLM treated group.
(TIF)

Figure S2 **Rupatadine attenuates the expression of inflammatory soluble factors in fibrotic lung tissue.** (**A**) Rupatadine (6.0 mg/kg per day) reduced the concentration of inflammatory cytokines in the BALF. The data are the representative results of two independent animal experiments (n=8) and expressed as the mean ± SEM of 3 assays with triplicates. (**B**) Rupatadine treatment decreased the expression of inflammatory cytokines in fibrotic mice lung tissue. Lung tissue extracts were prepared and the expression of inflammatory cytokines was detected by western blotting. The data are representative immune blots and quantified as the mean ± SEM of 3 independent assays. (**C**) Rupatadine (6.0 mg/kg per day) regulated the expression of crucial transcription factors in fibrotic lung tissue. The data are representative immune blots and are quantified as the mean ± SEM of 3 independent assays (n=6 mice per group). ^#^ *P* <0.05, ^##^ *P*<0.01 vs. Sham group; ^*^ *P*<0.05, ^**^ *P*<0.01 vs. BLM treated group.
(TIF)

Figure S3 **Rupatadine attenuates silica-induced chronic inflammation and pulmonary fibrosis.** Intratracheally administered SiO2 (100 mg/kg), the rats were intragastrically administered solvent only (sham group), or rupatadine at 1, 2 and 4 mg/kg from day 60 to 90. On day 90 the rats were sacrificed and a lung was obtained for histological analysis and other examination. (A) Rupatadine attenuated silica induced inflammation and decreased the number of silicon nodes and collagen deposition. The data are representative H&E staining (top) and Sirius Red (SR) staining (bottom) from three assays with identical results. Scale bar in images = 200 μm. (B) Rupatadine improved lung function in silica-induced fibrotic rats. Rupatadine reduced silica-enhanced airway dynamic resistance (left panel) and dynamic elasticity (middle panel), attenuated silica-induced low dynamic lung compliance (right panel). (C) Rupatadine treatment decreased TGF-β1 level in the BALF of fibrotic rats. The data are expressed as the mean ± SEM of 3 assays with triplicates. (D) Rupatadine decreased content of hydroxyproline and the expression of α-SMA in fibrotic lung tissue. The data for hydroxyproline are expressed as the mean ± SEM of 3 assays with triplicates. The data for α-SMA expression are representative immune blots and are quantified as the mean ± SEM of 4 assays with duplicates. ^#^ *P*<0.05, ^##^ *P*<0.01 vs. Sham group; ^*^ *P*<0.05, ^**^ *P*<0.01 vs. silica treated group.
(TIF)
