# Supplementary figures and images for: Rupatadine Protects against Pulmonary Fibrosis by Attenuating PAF-Mediated Senescence in Rodents
Source: PLoS One. 2013 Jul 15;8(7):e68631. doi: 10.1371/journal.pone.0068631 (PMC3711902; doi:10.1371/journal.pone.0068631)

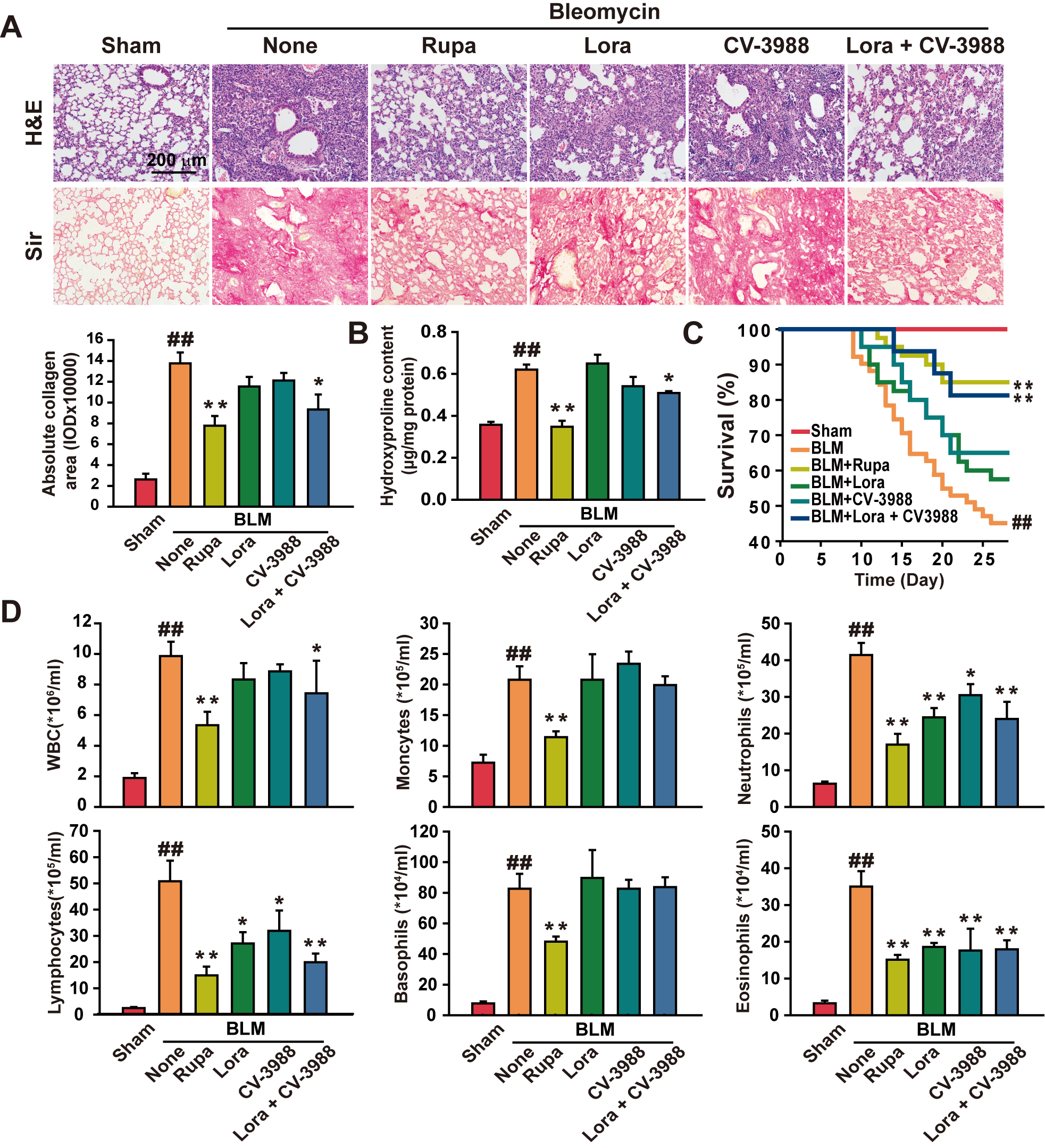

Supplement: Figure S1 — Anti-fibrotic effect of rupatadine is superior to H1 antagonist loratadine and PAF antagonist CV-3988. After intratracheally administered BLM (5 U/kg), the mice were intragastrically administered solvent only (Sham group), rupatadine (3.0 mg/kg), loratadine (3.0 mg/kg), CV-3988 (3.0 mg/kg), or 3.0 mg/kg loratadine plus 3.0 mg/kg CV-3988 (Lora + CV-3988) from day 10 to 28. On day 28, the mice were sacrificed and a lung was obtained for histological analysis and other examination. (A) Rupatadine treatment had the best anti-inflammation and anti-collagen deposition effects after BLM-injured. Histological examination was performed by hemotoxylin–eosin (H&E) staining (A, top) and Sirius Red (SR) staining (A, bottom). Scale bar in images = 200 μm. (B) Rupatadine and drug combination regimen reduced the hydroxyproline contents in fibrotic mice lung tissue. The data are expressed as the mean ± SEM of 10 mice per group. (C) Rupatadine and drug combination regimen significantly elevated survival rate of BLM-injured mice (n=40 per group which were at the start of the experiment). (D) BALF was collected on day 28 and classes of leukocytes quantified by differential counting. Rupatadine significantly inhibited the recruitment of inflammatory cells. The data are expressed as the mean ± SEM of 8 mice per group. # P<0.05, ## P<0.01 vs. Sham group; * P<0.05, ** P<0.01 vs. BLM treated group. (TIF) [file pone.0068631.s001.tif]

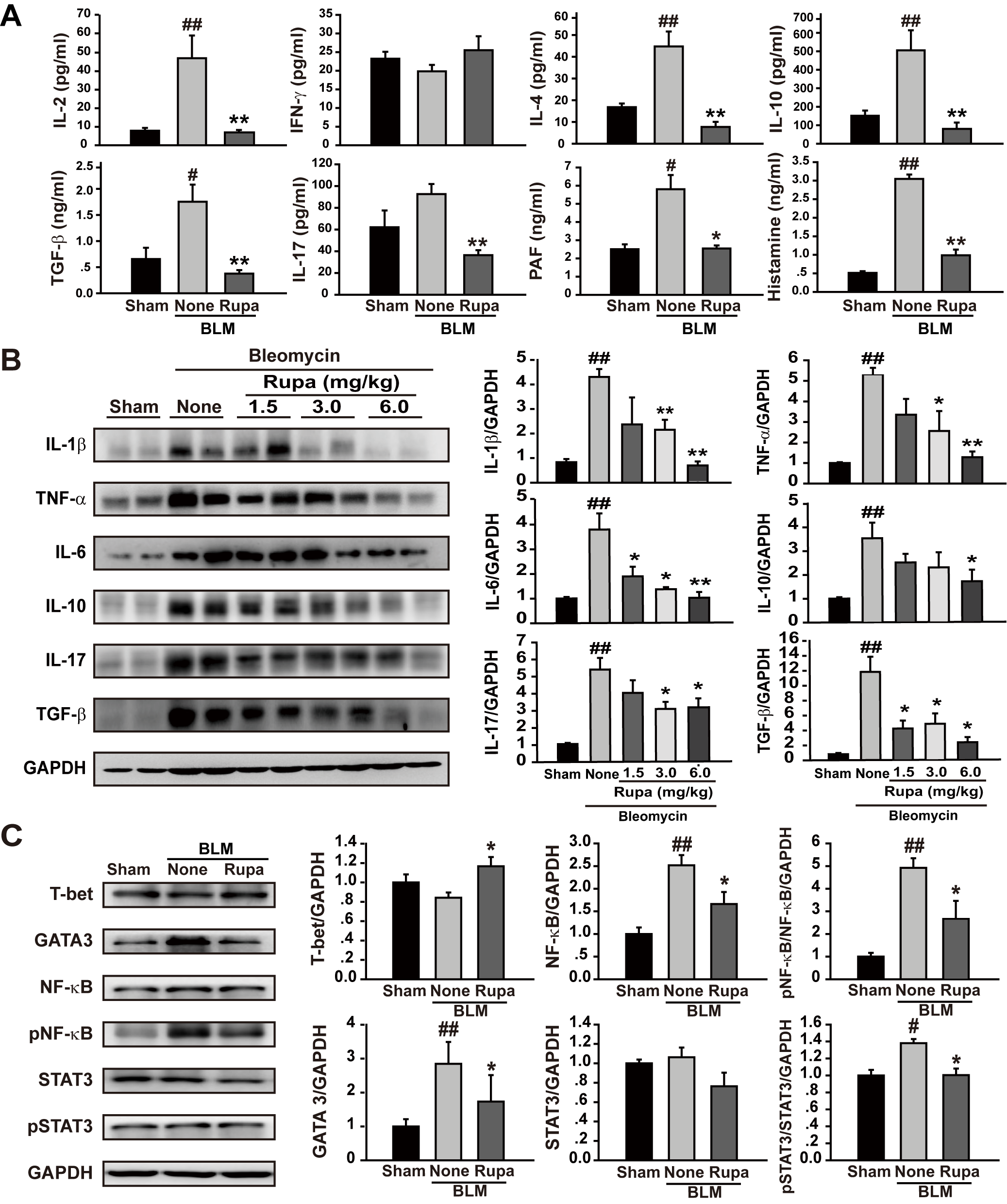

Supplement: Figure S2 — Rupatadine attenuates the expression of inflammatory soluble factors in fibrotic lung tissue. (A) Rupatadine (6.0 mg/kg per day) reduced the concentration of inflammatory cytokines in the BALF. The data are the representative results of two independent animal experiments (n=8) and expressed as the mean ± SEM of 3 assays with triplicates. (B) Rupatadine treatment decreased the expression of inflammatory cytokines in fibrotic mice lung tissue. Lung tissue extracts were prepared and the expression of inflammatory cytokines was detected by western blotting. The data are representative immune blots and quantified as the mean ± SEM of 3 independent assays. (C) Rupatadine (6.0 mg/kg per day) regulated the expression of crucial transcription factors in fibrotic lung tissue. The data are representative immune blots and are quantified as the mean ± SEM of 3 independent assays (n=6 mice per group). # P <0.05, ## P<0.01 vs. Sham group; * P<0.05, ** P<0.01 vs. BLM treated group. (TIF) [file pone.0068631.s002.tif]

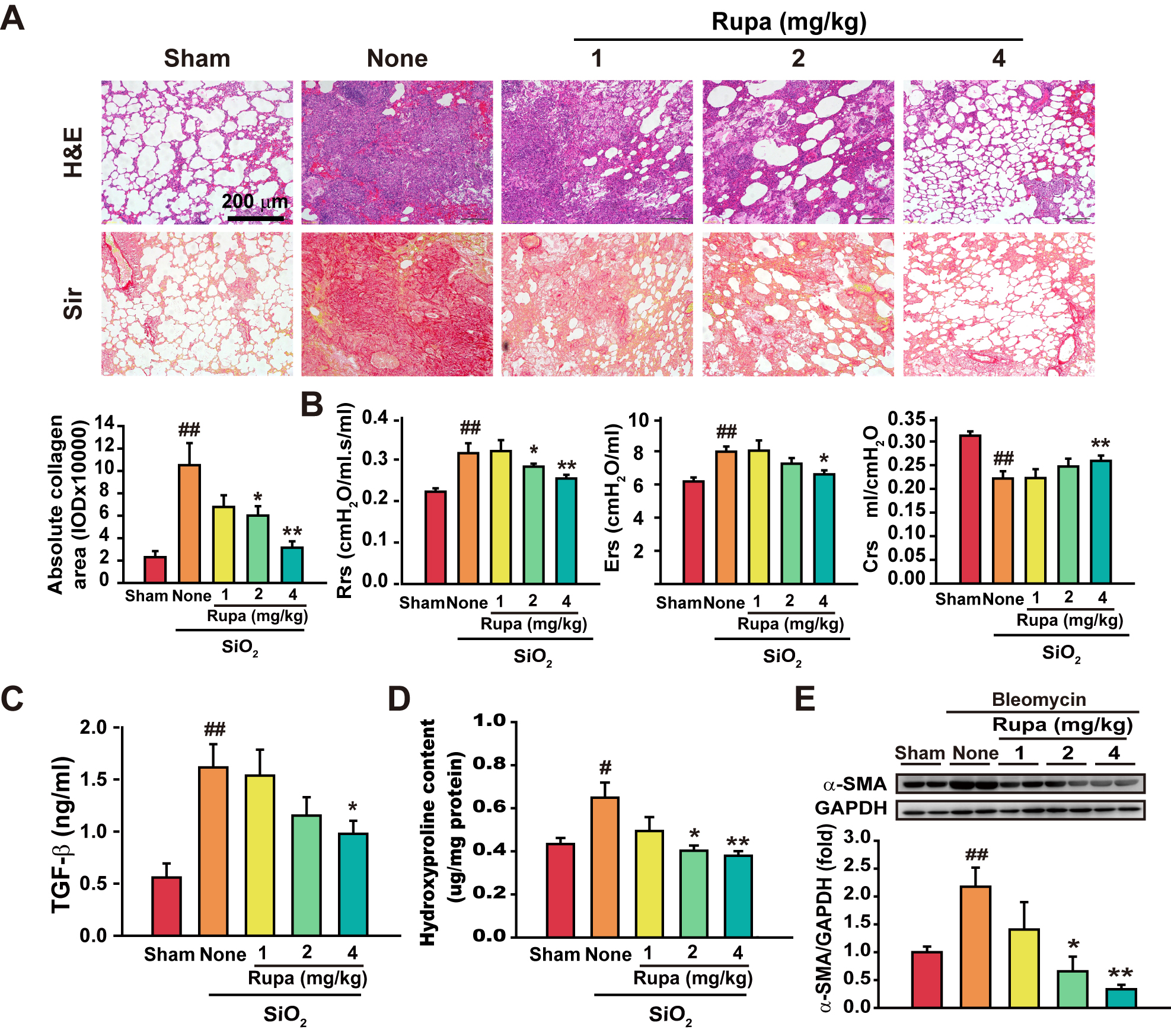

Supplement: Figure S3 — Rupatadine attenuates silica-induced chronic inflammation and pulmonary fibrosis. Intratracheally administered SiO2 (100 mg/kg), the rats were intragastrically administered solvent only (sham group), or rupatadine at 1, 2 and 4 mg/kg from day 60 to 90. On day 90 the rats were sacrificed and a lung was obtained for histological analysis and other examination. (A) Rupatadine attenuated silica induced inflammation and decreased the number of silicon nodes and collagen deposition. The data are representative H&E staining (top) and Sirius Red (SR) staining (bottom) from three assays with identical results. Scale bar in images = 200 μm. (B) Rupatadine improved lung function in silica-induced fibrotic rats. Rupatadine reduced silica-enhanced airway dynamic resistance (left panel) and dynamic elasticity (middle panel), attenuated silica-induced low dynamic lung compliance (right panel). (C) Rupatadine treatment decreased TGF-β1 level in the BALF of fibrotic rats. The data are expressed as the mean ± SEM of 3 assays with triplicates. (D) Rupatadine decreased content of hydroxyproline and the expression of α-SMA in fibrotic lung tissue. The data for hydroxyproline are expressed as the mean ± SEM of 3 assays with triplicates. The data for α-SMA expression are representative immune blots and are quantified as the mean ± SEM of 4 assays with duplicates. # P<0.05, ## P<0.01 vs. Sham group; * P<0.05, ** P<0.01 vs. silica treated group. (TIF) [file pone.0068631.s003.tif]
